# Cost-effectiveness-analysis of ultrasound guidance for central venous catheterization compared with landmark method: a decision-analytic model

**DOI:** 10.1186/s12871-019-0719-5

**Published:** 2019-04-09

**Authors:** Yana Seleznova, Patrick Brass, Martin Hellmich, Stephanie Stock, Dirk Müller

**Affiliations:** 1 Institute for Health Economics and Clinical Epidemiology, The University Hospital of Cologne (AöR), Gleueler Str. 176-178, 50935 Cologne, Germany; 2 Department of Anaesthesiology, Intensive Care Medicine, and Pain Therapy, Helios Hospital Krefeld, Lutherplatz 40, 47805 Krefeld, Germany; 3 Institute of Medical Statistics and Computational Biology, University of Cologne, Bachemer Str. 86, 50931 Cologne, Germany

**Keywords:** Cost-effectiveness, Ultrasound guidance, Central venous catheterization, Modelling

## Abstract

**Background:**

Ultrasound guidance for central venous catheterization is a commonly used alternative to the conventional landmark method. Because from the German perspective, the cost-effectiveness of ultrasound guidance is unclear, this study examined the cost-effectiveness of ultrasound guidance versus the landmark method for adults undergoing a central venous catheterization.

**Methods:**

A decision-tree based model was built to estimate the costs of averted catheter-related complications. Clinical data (e.g. arterial puncture, failed attempts) were obtained from a Cochrane review and a randomized controlled trial, whilst information about cost parameters were taken from a German hospital of maximum care. The analysis was conducted from the perspective of the German Statutory Health Insurance. Results were presented as incremental cost-effectiveness ratios. To assess the parameter uncertainty, several sensitivity analyses were performed (deterministic, probabilistic and with regard to the model structure).

**Results:**

Our analysis revealed that ultrasound guidance resulted in fewer complications per person (0.04 versus 0.17 for the landmark method) and was less expensive (€51 versus €230 for the landmark method). Results were robust to changes in the model parameters and in the model structure. Whilst our model population reflected approximately 49% of adults undergoing a central venous catheterization cannulation per year, structural sensitivity analyses (e.g. extending the study cohort to patients at higher baseline risk of complications, pediatric patients, or using real-time/indirect catheterization) indicated the cost-effectiveness of ultrasound guidance for a broader spectrum of patients. The results should be interpreted by considering the assumptions (e.g. target population) and approximations (e.g. cost parameters) underpinning the model.

**Conclusions:**

Ultrasound guidance for central venous catheterization averts more catheter-related complications and may save the resources of the German Statutory Health Insurance compared with landmark method.

**Electronic supplementary material:**

The online version of this article (10.1186/s12871-019-0719-5) contains supplementary material, which is available to authorized users.

## Introduction

Central venous catheterization enables diagnostic and therapeutic purposes in anesthesia and intensive care. It is practiced for various clinical reasons such as for the infusion of potent or irritating drugs (e.g. vasopressors), for the administration of fluids and for hemodynamic monitoring (e.g. oxygen saturation) [1–3]. Central venous catheters are also used in other disciplines such as nephrology or cardiology. They are needed for hemodialysis or for the placement of pacemakers during a cardiac catheterization [4].

Among different methods to perform a central venous catheterization, the conventional landmark method (LM) is the one most commonly applied in anesthesia and intensive care units. Using this technique, the insertion is guided by the surface anatomical structures but may lead to different complications such as arterial puncture, pneumothorax or failed insertions [5]. According to the clinical literature, complication rates and failure rates of the LM amount to 19% [6].

To reduce the complications associated with a central venous catheterization, different puncture sites, puncture techniques and, the use of ultrasound imaging were considered as alternatives for access [3]. Especially the ultrasound guidance (UG) for central venous catheterization is advanced and its efficacy was evaluated in various trials [7–9]. Sonographic techniques refer to different ultrasound modalities such as ultrasound Doppler and two-dimensional ultrasound technique. The two-dimensional ultrasound is more commonly used for central venous catheter placement in the German health care setting [5]. This placement can be performed indirectly or, directly and real-time, respectively. A real-time cannulation enables to visualize the target vein and surrounding anatomical structures before and during the procedure. In contrast, by choosing an indirect catheterization, the ultrasound is used only for improving vessel orientation before the puncture. The cannulation itself is performed without the UG [3, 5].

A recent Cochrane review showed that the use of real-time two-dimensional ultrasound increases the chance of a successful cannulation on the first attempt by 58% and reduces the rate of total complications significantly by 67% compared with LM [3]. Because of the higher efficacy of ultrasound guided catheterization (i.e. the lower rate of complications and failed cannulations), international and national guidelines recommend UG for patients undergoing a central venous catheterization [1, 2].

International studies have analyzed the cost-effectiveness of UG versus LM and have shown the UG to be a cost-saving catheterization strategy [10, 11]. In Germany more than a half million central venous catheters are placed annually [12], whilst evidence on economic impacts of using UG is still lacking. The aim of this analysis was to assess the costs per avoided complication of the real-time two-dimensional UG compared to traditional LM in German adults undergoing a central venous catheterization. We performed this model-based cost-effectiveness analysis from the perspective of the German Statutory Health Insurance (SHI).

## Methods

### Patient and interventions

The target population of the model is aligned on the population of a recent Cochrane review [3]. It represents a cohort of patients aged ≥18 years who undergo a central venous catheterization. In Germany, about 96% (*n* = 548,000) of central catheters are provided to patients ≥18 years [12]. Because 70% (*n* = 384,000) of central catheters are placed via the internal jugular vein (IJV) [5] and, thereby simultaneously 70% in real-time catheterization, [3] our model population reflects approximately 49% (*n* = 269,000) of adults undergoing a central venous catheterization per year.

Patients received a central venous catheterization using either UG or LM. UG was assumed to be performed by the real-time catheterization. In addition, both UG and LM are inserted via the IJV in the Seldinger technique.

### Model description

**Fig. 1:**
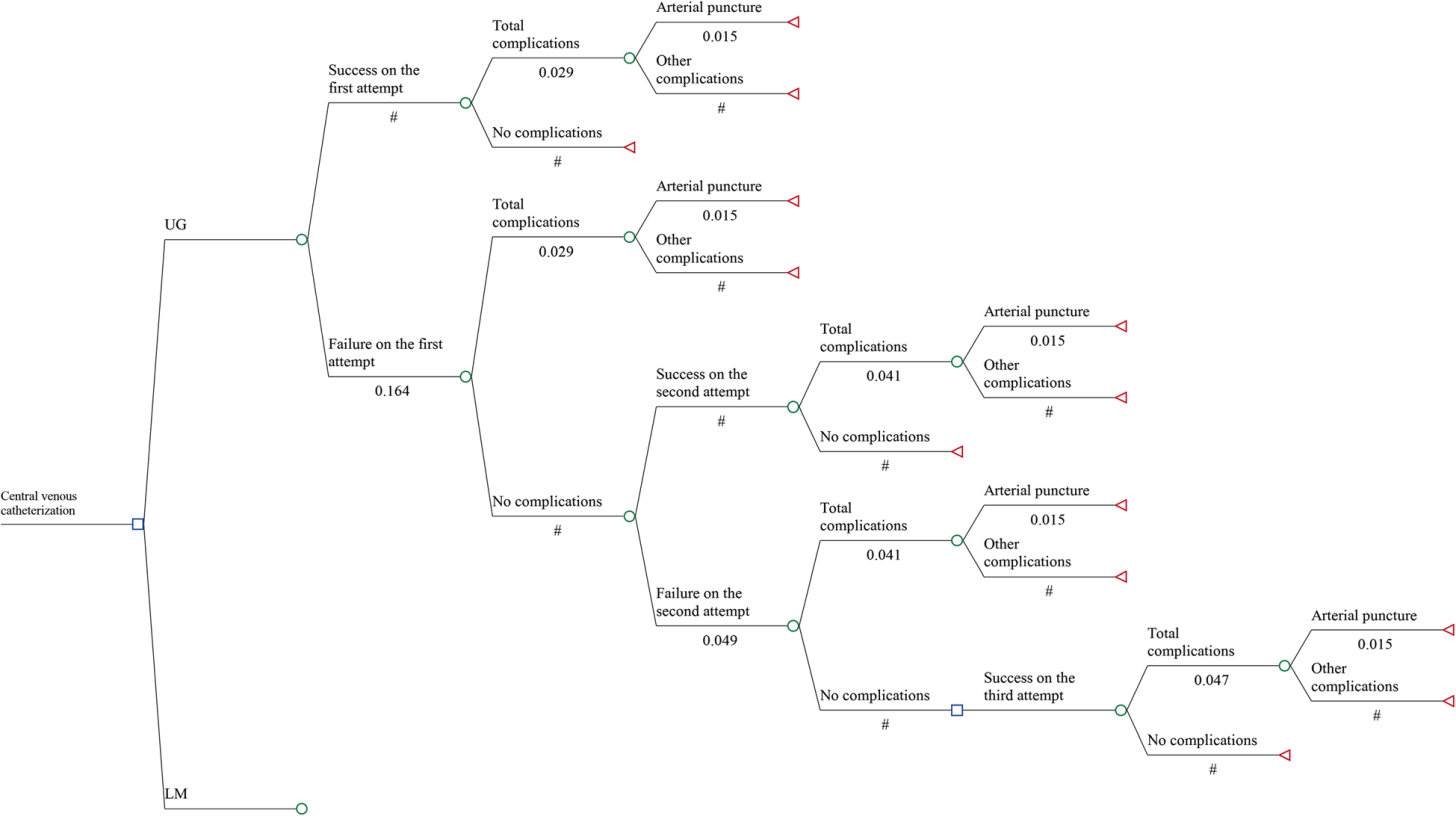
Decision-tree based model: health states and outcomes during an ultrasound guided cannulation. *UG* Ultrasound guidance, *LM* Landmark method, *#* Complementary probability 1-p. The pathway of landmark method has the same structure

We constructed a decision-tree based model [13] to reflect the current practice and the clinical pathway of a central venous catheterization performed by either UG or LM. Figure 1 is a schematic representation of the model showing the pathway of UG and, the starting point of LM which has the same structure. A hypothetic cohort of patients undergoing a central venous catheterization in UG or LM (Fig. 1) may experience failed attempts of catheterization and catheter-related complications. For UG and LM, the model considered the likelihood of cannulation failure and, with regard to clinical trials; we assumed maximum three attempts to be required for a successful cannulation [8, 14]. Both a successful and a failed catheterization may result in different complications (Fig. 1). Since complication rates increase significantly with the number of attempts [9], we considered this increase in the model. Complications were reflected by two states in the model; ‘arterial puncture’ and ‘other complications’. The state ‘arterial puncture’ was chosen because it is the most often reported complication with rates between 1.6 and 3% for the UG and between 8.5 and 12% for the LM [3, 15, 16]. Due to the overall low rate (0.42% UG/ 2.21% LM) of other complications [3, 15, 16] these are summarised in a composite endpoint. The state ‘other complications’ comprises thrombosis, embolism, hydromediastinum, haematomaediastinum, hematothorax, hydrothorax, pneumothorax, nerve injury, and subcutaneous emphysema.

Health outcomes were presented as the number of averted complications because data [3, 15, 16] on perioperative mortality, health-related quality of life or catheter-related infections were not available. To estimate the averted complications, an event was coded as ‘0’ and no event as ‘1’. The time horizon of the model covered a period of 1 year because clinical and monetary consequences resulting from a central venous catheterization beyond this time are unlikely [10, 11]. The model was built and calculated with TreeAge Pro 2017© (Williamstown, Massachusetts).

### Clinical data

The probabilities of catheter-related complications and failed attempts which were included into the decision-analytic model (Fig. 1) are based on a recent Cochrane review [3]. In this systematic review, efficacy and safety of real-time two-dimensional UG was evaluated in comparison to LM for insertion of central venous catheters via the internal jugular vein in adults. We obtained the rates of failure on the first and second attempt, total complications, arterial puncture, and other complications from meta-analyses included in the Cochrane review [3]. All rates were transformed into probabilities corresponding to the time horizon of the model (1 year) [17]. The increased risk for catheter-related complications for repeated attempts was based on a single randomized controlled trial [9]. Compared to the first attempt, the odds ratio of suffering a complication during the second and third attempt is 8.4 (*p* < 0.001) and 35.6 (*p* < 0.001), respectively. Table 1 summarizes the clinical input data which were included into the model.

**Table 1:** Clinical input data

Ultrasound guidance	Probabilities of events (95% CI)	Reference
Failure on the first attempt	0.164 [0.143, 0.183]	[3]
Failure on the second attempt	0.049 [0.031, 0.067]	[3]
Total complications on the first attempt	0.029 [0.019, 0.040]	[3]
Total complications on the second attempt	0.041 [0.029, 0.053]	[3, 9]
Total complications on the third attempt	0.047 [0.034, 0.059]	[3, 9]
Arterial puncture^a^	0.015 [0.010, 0.021]	[3]
Landmark method		
Failure on the first attempt	0.376 [0.356, 0.395]	[3]
Failure on the second attempt	0.166 [0.138, 0.193]	[3]
Total complications on the first attempt	0.114 [0.096, 0.132]	[3]
Total complications on the second attempt	0.156 [0.136, 0.176]	[3, 9]
Total complications on the third attempt	0.177 [0.156, 0.197]	[3, 9]
Arterial puncture^a^	0.081 [0.069, 0.093]	[3]

*CI* confidence interval

^a^The probabilities of an arterial puncture relate to each additional attempt

### Cost data

Parameters of direct medical costs were based on claims data from 2015, which were used for reimbursement purposes of providers [13, 18] with the German SHI. To reflect the cost structure of the treatment alternatives, all data on costs were obtained from the German Helios Hospital Krefeld [19]. This hospital of maximum care was assumed to be representative for the spectrum of patients receiving a central venous catheterization and, the coding practices for reimbursement. All costs were given in €2016 values. If data from previous years were used, these were adjusted for inflation [20] with respect to the German harmonised index of consumer prices [21] for inpatient care services [22]. According to recommendations by the German Institute for Quality and Efficiency in Health Care (IQWiG) [21], we did not discount the costs and benefits because they relate to a period of 1 year.

#### Identification of relevant costs

The identification of costs from the perspective of the SHI was aligned on the clinical pathway reflected by the decision tree (Fig. 1). We included treatment costs of catheter-related complications as they are charged to the SHI. Costs resulting from the resource use of the equipment for an additional attempt of insertion and intervention cost of UG were excluded from the analysis because they are not reimbursed by the SHI. In particular, the intervention costs of UG comprise purchase costs, maintenance costs of equipment, costs for disposable equipment (e.g. ultrasound probes sheaths) and costs for training staff (Additional file 1). These costs are borne by hospitals applying the UG. Further, costs incurring to patients or families as well as productivity costs were not estimated as they do not accrue for the SHI-perspective [21].

#### Measurement and valuation of complications costs

Treatment costs of catheter-related complications were based on diagnosis-related groups (DRGs) [23]. These costs were estimated in accordance with the International Classification of Diseases (ICD), 10. Revision, German Modification [24] and, by calculating with the DRG-Grouper.

The amount of costs of a specific DRG depends on different factors such as the nature and extent of related procedures, secondary diagnoses and the principal ICD-Diagnosis [25]. According to the coding practices [23, 24], the costs of complications were approximated using the secondary diagnosis. The approximation was run on the hospital database (*n* = 55,000) and calculated for both the costs with and without the corresponding ICD-complications. The resulting difference was assumed to reflect the treatment costs of catheter-related complications (Table 2).

**Table 2:** Cost input data from the perspective of the German Statutory Health Insurance

Variable	Cost per complication in €	Reference
Complications		
Arterial puncture	94	[19]
Thrombosis	131	
Embolism	131	
Hydromediastinum	133	
Hematomediastinum	94	
Hematothorax	177	
Hydrothorax	217	
Pneumothorax	178	
Nerve injury	347	
Subcutaneous emphysema	16	

### Sensitivity analyses and model validation

Deterministic and probabilistic sensitivity analyses [17] were undertaken to evaluate the impact of the variation of input data and assumptions made for the model. To identify the parameters with the largest impact on the baseline results, we run an univariate deterministic analysis for all input parameters. Confidence intervals from clinical data were used for the variation of the probability of complications (Table 1). Due to a lack of data, we varied cost parameters by ±50% which is in line with the recommendation for handling uncertainty in economic evaluations. In detail, it is recommended to vary parameters by a specified amount (e.g. plus or minus a proportional change in its mean value) [13]. In addition, a probabilistic sensitivity analysis using a Monte-Carlo-simulation with 10,000 iterations was performed to model a simultaneous change of model parameters (Additional files 2 and 3). The parameters for distributions were approximated based on the corresponding expected value and the standard error. We defined beta distributions for probabilities and gamma distributions for costs.

Additionally, to account for dependence of cost-effectiveness results on the included patient population (i.e. uncertainty inherent to the model structure) [17], we performed structural sensitivity analyses by broadening the patient population. To reflect the cost-effectiveness of UG in patients with a higher baseline risk (e.g. coagulopathic patients [1, 26], patients with anatomic deformities or difficult veins [26, 27]) – who were not regularly included in clinical trials - we calculated a hypothetical scenario by doubling or tripling the baseline rates (Table 1) of complications. Further we applied analyses including pediatric patients and/or an indirect UG based on the meta-analyses from the Cochrane review [3].

To validate the model [28], clinical experts involved in the analysis evaluated how well each model component reflected their understanding of the pertinent medical science and the available evidence (face validity). We discussed the model structure (Fig. 1), the functional relationships in the model and, the clinical evidence used throughout the model development. For example, the option of several catheterization attempts was consented to be appropriate because of the increased risk for complications. In order to ensure that the model is implemented as intended, estimates of cost data were verified by accounting experts of the German Helios Hospital Krefeld [19] (technical validity). Furthermore, we compared the model structure and results of this model to that of available cost-effectiveness models [10, 11] from the UK and Brazilian health care systems in this field (cross-validation).

### Budget impact analysis

A budget impact analysis was performed to address the potential expected additional costs or savings for the SHI which might result from an adoption of UG to central venous catheterization [29]. To quantify the annual burden on the SHI, the total additional costs of using UG (i.e. costs resulting from intervention strategy minus costs resulting from control strategy) were multiplied by the expected number of ultrasound governed central venous catheterizations per year [12]. Providing that UG for central venous catheterization is added to the conventional LM technique and both interventions are used [29], we conservatively assumed that 10 to 20% of annually placed central venous catheters (*n* = 568,000) are placed with the UG.

## Results

For the base-case, the utilisation of UG for a central venous catheterization reduces the costs of a cannulation by €179 per procedure compared to the LM. The use of UG is associated with on average 0.14 less complications. Therefore, from the perspective of the German SHI, UG dominates the LM and is the preferred strategy (i.e. less costly and more effective, Table 3).

**Table 3:** Incremental cost-effectiveness of ultrasound guidance versus landmark method (base-case)

Strategy	Costs (€)	Incremental costs (€)	Complications per person	Incremental effect (averted complications per person)	ICER (€ per averted complication)
Ultrasound guidance	51	−179	0.036	0.138	Dominates^a^
Landmark method	230		0.175		

*ICER* incremental cost-effectiveness ratio

^a^Ultrasound guidance is less costly and more effective in averting complications compared with landmark method

Deterministic sensitivity analysis (Additional file 4) shows that the cost-effectiveness of the UG is most sensitive to the costs for the treatment of the composite endpoint ‘other complications’. Doubling or halving the treatment costs of ‘other complications’ reduces/increases the incremental cost-effectiveness ratio by ±12% for the costs of ‘nerve injury’ and ± 3% for the costs of ‘haematomediastinum’. The clinical parameter with the largest impact on the cost-effectiveness is the probability of an arterial puncture using LM (±2%). Increasing or decreasing other variables changed the incremental cost-effectiveness ratio by less than 2%.

**Fig. 2:**
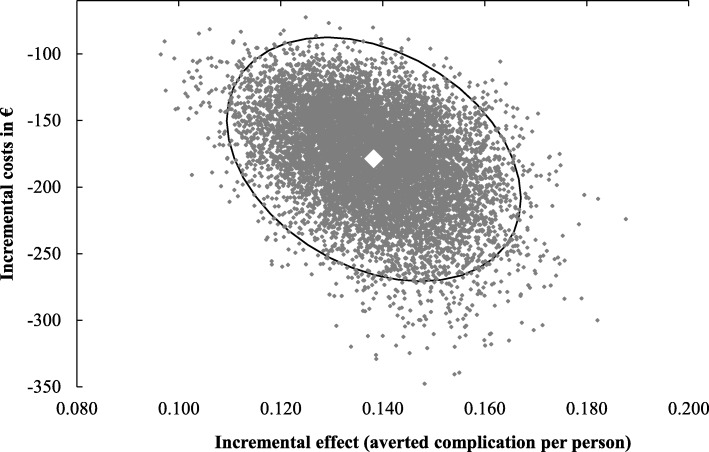
Results of the probabilistic sensitivity analysis showing the distributions of costs and effects for UG. *UG* Ultrasound guidance. ◊, base-case

The base-case results were robust in the probabilistic sensitivity analysis (Fig. 2). In particular, performing a simultaneous variation of all input parameters, the UG dominates the LM in all iterations. Comparing the alternatives in terms of costs, the upper limit of the 95% CI €78 of the UG is lower than the lower limit €151 of the LM. In terms of efficacy, the lower limit of the 95% CI of effectiveness (0.95) is higher than the higher limit (0.85) of the LM. Structural sensitivity analyses confirmed the results for different alternatives (Table 4).

**Table 4:** Results of the structural sensitivity analyses

Strategy	Costs (€)	Incremental costs (€)	Complications per person	Incremental effect (averted complications per person)	ICER (€ per averted complication)
Alternative 1: Doubled complication rates, adults and real time-cannulation					
Ultrasound guidance	141	− 277	0.076	0.267	Dominates^a^
Landmark method	418		0.343		
Alternative 2: Tripled complication rates, adults and real time-cannulation					
Ultrasound guidance	203	− 449	0.123	0.457	Dominates^a^
Landmark method	652		0.579		
Alternative 3: Paediatric and adult patients, real-time cannulation					
Ultrasound guidance	70	− 161	0.050	0.127	Dominates^a^
Landmark method	232		0.177		
Alternative 4: Paediatric and adult patients, real-time and indirect cannulation					
Ultrasound guidance	68	− 185	0.049	0.145	Dominates^a^
Landmark method	253		0.194		

*ICER* incremental cost-effectiveness ratio

^a^Ultrasound guidance is less costly and more effective in averting complications compared with landmark method

Assuming a higher baseline risk by doubling the complications rates would result in 0.27 (Alternative 1) and by tripling in 0.46 (Alternative 2) less complications per person compared to LM. In these alternatives UG remains less costly than LM. The use of UG in both adult and pediatric patients in real-time only (Alternative 3) resulted on average in 0.13 less complications and was also less costly compared to LM. Similarly, the UG in pediatric and adult patients cannulated in real-time and indirect cannulation (Alternative 4), is associated with on average 0.15 less complications. Even a simultaneous variation of all input parameters (i.e. probabilistic sensitivity analysis of alternatives 3–4) in the structural sensitivity analyses did not change (Additional file 5) the main cost-effectiveness results of UG.

Based on our budget impact analysis for the base-case scenario the use of UG for a central venous catheterization could avoid 8000 to 16,000 complications each year and might result in annual cost savings from €10.2 to €20.3 million for the SHI.

## Discussion

This model-based analysis is the first study which examined the cost-effectiveness (costs of averted catheter-related complications) of ultrasound guided versus landmark oriented central venous catheterization in German adults. In the base case, the use of UG for a central venous catheterization compared with LM reduces the costs by €179 per procedure and is associated with on average 0.14 less complications. In line with previous studies from other health systems [10, 11], our results confirm, the use of UG for central venous catheterization being more effective in averting complications and less costly than the LM. These results did not change when various sensitivity analyses were applied. Replacing LM by UG could result in at least 8000 avoided complications annually which might correspond to a minimum of cost savings of €10.2 million each year for the German SHI.

Our model has several strengths. First, the analysis considers the correlation between the number of cannulation attempts and the total complication rate. Incorporating the increased likelihood of a complication in relation to the number of cannulation attempts enables a more realistic reflection of the clinical pathway of a central venous catheterization. Unfortunately, the Cochrane review [3] and the underlying studies do not specify whether the number of arterial puncture was measured on the first or second attempt. However, results of the univariate sensitivity analyses showed that a variation of the incidence of arterial puncture affects the cost-effectiveness ratio by ≤2%, i.e., UG would be more effective and less costly.

Second, the cost estimation of catheter-related complications was based on the recently used claims data from a hospital of maximum care (*n* = 55,000) which can be assumed to reflect the usual reimbursement practice of German hospitals. Using data from this hospital enabled us to estimate and include the costs of rare events [30] such as nerve injury or pneumothorax.

Third, the costs of treatments for complications were calculated with respect to regulation principles and specific features of the German DRG-System. More specifically, rules of coding, grouping and accounting for complications were taken into account [18, 23–25].

Finally, the target population of the model was in line with patients included in the Cochrane-review [3] where we took clinical data. In addition, the chosen time frame of the analysis is appropriate to reflect the clinical consequences of UG.

In contrast, modelling studies tend to exhibit weaknesses because of constraints of resources and information availability. In our analysis, to some extent, the use of claims data for estimating several cost parameters might lead erroneous results [30]. For example, the measured catheter-related complications might be subject to up-coding, i.e. the severity or number of complications is increased to improve reimbursement [31]. Therefore, it cannot be excluded that the costs calculated for catheter-related complications were overestimated to some degree. To avoid an overestimation of costs we validated our model with experts. In addition, for the sensitivity analyses a conservative range of ±50% was used for handling uncertainty [13]. Supposed that these costs are still overestimated, the incremental cost-effectiveness ratio would be biased towards a cost saving impact of UG.

With regard to the input data of the model, there are some limitations resulting from the clinical evidence used for the model that may call our results into question. According to the authors of the Cochrane review [3] the internal validity of the included trials was insufficient due to several methodological deficiencies. In most of the studies, blinding of participants, operators and assessors were judged as unclear. Whilst personnel blinding was not possible due to the nature of intervention, a possible blinding of the outcome assessors and the patients would have reduced the risk of bias. In addition, in 80% of the included studies random sequence generation and allocation concealment was considered to be at moderate or high risk of bias.

Furthermore, some meta-analyses calculated in the Cochrane review were affected by study heterogeneity which may result from different sources. In particular, the meta-analyses for the success rates of the cannulation showed a substantial heterogeneity (> 50%). A qualitative examination conducted by the authors of the review revealed differences in the study populations, technical and methodological differences as potential reasons for this heterogeneity [3]. Specifically, a successful central venous catheterization depends on the operator’s expertise in cannulation. In studies of the Cochrane review, [3] the ‘learning curve’ of the operators varied from high-expertise practitioners [7] to operators with limited experience [26]. Because of the lack of information, we could not assess the impact of the practitioners’ expertise on the cost-effectiveness of UG.

The external validity of our study may be called into question because information about the number of excluded patients in clinical studies of UG and the reasons for their exclusion are sparse [7, 26, 32, 33]. Since in the base-case we had to rely on the inclusion criteria of the clinical studies included in the Cochrane review, the patient population in clinical practice might be at higher risk of complications. These complications might be affected by various patient characteristics such as anatomic variations, comorbidities or age. Anatomic variations such as the position or/and diameter (< 7 mm) of the IJV may complicate a central venous catheterization. For example, in 54% of the adults the IJV lies over the carotid artery [34] or, in 13–18% of individuals the IJV is about 5 mm [4]. If we assume that in 54% [34] of the adults (> 40 years) the IJV lies over the carotid artery, then our model would only refer to approximately 22% (*n* = 124,000) of the patients catheterized per year. Additionally, different comorbidities such as coagulative disorders, uremia or obesity may make a cannulation more difficult [3, 7]. To assess the external validity of our study we undertook several sensitivity analyses. The base-case results did not change when a study population with higher incidence for complications was assumed in structural sensitivity analyses (i.e. UG was still more effective in averting complications and less costly). Moreover, the use of UG averted even more complications (i.e., increase of 200% by tripled complication rates) compared to the base-case results.

Similarly, central venous catheterization is a challenging procedure in the pediatric population [1]. Various anatomic anomalies or a large range in the size of vessels may complicate a central cannulation compared to adults [35]. For example, 18% (*n* = 50) of patients < 6 years had anomalous venous anatomy that may account for some difficulties in the catheterization [33]. However, including both adults and children in the study population did not change the overall conclusions on clinical and economic benefits of UG.

Furthermore, in accordance with clinical studies, in our analysis central venous catheterization was assumed to be performed by the real-time ultrasonography and via the IJV. A scenario of patients catheterized by real-time and indirect technique did not alter the conclusions of the analysis and would even improve the results in favor of the UG.

In addition, the model assumed a central venous catheterization to be performed in intensive care or an operating theatre environment. It goes beyond the scope of this study to judge whether the conclusions derived are valid for emergency departments, where catheterizations might also be performed [3].

Since judging external validity is often more a clinical than a methodological expertise, future studies should be designed and reported in a way which allows clinicians to judge to whom they can reasonably be applied [36]. Thus, we aimed to report the determinants of our model and of the evidence which it is based on to allow clinicians and other decision makers to reach their conclusions.

Despite some limitations which might affect the cost-effectiveness of UG, our results are in line with analyses [10, 11] from other health systems. In contrast to previous analyses, our analysis considers more catheter-related complications (e.g. pneumothorax, nerve injury) resulting in more averted complications compared to the results of other models. With respect to the expected costs of a central venous catheterization, our analysis confirms the cost saving potential of the UG in other studies. Nevertheless, there are some differences in the included costs, in particular the costs of UG intervention (e.g. UG machines).

Relevant costs to be included depend on the taken perspective [20], especially on the reimbursement procedures in the setting. Because the costs of UG intervention are not charged to the SHI, we did not include them in our analysis. Supposed that the SHI would reimburse the costs of the intervention, UG would be still a cost saving option up to €179 per catheterization (Additional file 6). This amount is approximately fivefold of the estimated costs which are borne by the hospital considered in this analysis. Since relevant costs and their estimation are subject to country specific variation [37], the variation of cost savings between different countries is unavoidable.

In addition, recent evidence indicates advantages of UG over chest x-ray because UG can be performed faster and does not subject patients to radiation [38]. Including these costs may improve the cost-effectiveness result in favor of UG.

## Conclusions

In summary, the use of UG averts more catheter-related complications and may save the resources of the German SHI compared to the LM. Because the technological advance of ultrasound devices and capabilities in dealing with them will improve in the future, an increasing use of UG can be carefully advised.

## Additional files

Additional file 1: Intervention costs of an ultrasound guidance. These costs are borne by the hospital of maximum care and are not reimbursed by the SHI. (DOC 31 kb)

Additional file 2: Cost data input for deterministic and probabilistic sensitivity analyses. (DOC 34 kb)

Additional file 3: Clinical data input for the probabilistic sensitivity analysis (base-case). (DOC 37 kb)

Additional file 4: Tornado-diagram presenting the results of the univariate deterministic sensitivity analysis (i.e., top ten parameters with the greatest impact on incremental cost-effectiveness ratio). (PPTX 66 kb)

Additional file 5: Results of the structural sensitivity analyses presenting the output of a probabilistic parameter variation. (PPTX 620 kb)

Additional file 6: Results of the threshold analysis for intervention costs of ultrasound guidance. (PPTX 70 kb)

## Abbreviations

DRG: Diagnosis-Related Groups
ICD: International Classification of Diseases
ICER: Incremental cost-effectiveness ratio
IJV: Internal jugular vein
LM: Landmark method
SHI: Statutory Health Insurance
UG: Ultrasound guidance
