# Supplementary material for: Cost-effectiveness-analysis of ultrasound guidance for central venous catheterization compared with landmark method: a decision-analytic model
Source: BMC Anesthesiol. 2019 Apr 9;19:51. doi: 10.1186/s12871-019-0719-5 (PMC6456944; doi:10.1186/s12871-019-0719-5)
Supplement: Supplementary file 1 — Intervention costs of an ultrasound guidance. These costs are borne by the hospital of maximum care and are not reimbursed by the SHI. (DOC 31 kb) [file 12871_2019_719_MOESM1_ESM.doc]

**Additional file** **1:** Intervention costs of an ultrasound guidance borne by the hospital of maximum care

| **Costs per procedure in €** | |  | |  |  | **Reference** |
| --- | --- | --- | --- | --- | --- | --- |
|  | Purchase costs | |  |  | 23.63 | [19] |
|  | Maintenance costs of equipment | |  |  | 4.49 |  |
|  | Cost of disposable equipment  (UG probes sheath, UG gel) | |  |  | 5.77 |  |
|  | Training costs | |  |  | 2.13 |  |
| Total costs | |  | |  | 36.02 |  |

*UG* ultrasound guidance
