# Supplementary material for: Cost-effectiveness-analysis of ultrasound guidance for central venous catheterization compared with landmark method: a decision-analytic model
Source: BMC Anesthesiol. 2019 Apr 9;19:51. doi: 10.1186/s12871-019-0719-5 (PMC6456944; doi:10.1186/s12871-019-0719-5)
Supplement: Supplementary file 2 — Cost data input for deterministic and probabilistic sensitivity analyses. (DOC 34 kb) [file 12871_2019_719_MOESM2_ESM.doc]

**Additional file** **2:** Cost data input for deterministic and probabilistic sensitivity analyses

| **Variable** | **Cost per complication**  **in € (**± **50%)** | **Reference** |
| --- | --- | --- |
| Arterial puncture | 94 (47) | [19] |
| Thrombosis | 131 (66) |  |
| Embolism | 131 (66) |  |
| Hydromediastinum | 133 (67) |  |
| Hematomediastinum | 94 (47) |  |
| Hematothorax | 177 (89) |  |
| Hydrothorax | 217 (109) |  |
| Pneumothorax | 178 (89) |  |
| Nerve injury | 347 (173) |  |
| Subcutaneous  emphysema | 16 (8) |  |
