# Supplementary material for: Cost-effectiveness-analysis of ultrasound guidance for central venous catheterization compared with landmark method: a decision-analytic model
Source: BMC Anesthesiol. 2019 Apr 9;19:51. doi: 10.1186/s12871-019-0719-5 (PMC6456944; doi:10.1186/s12871-019-0719-5)
Supplement: Supplementary file 3 — Clinical data input for the probabilistic sensitivity analysis (base-case). (DOC 37 kb) [file 12871_2019_719_MOESM3_ESM.doc]

**Additional file 3: Clinical data input for the probabilistic sensitivity analysis (base-case)**

| **Variable** | **Mean (SE)** | **Reference** |
| --- | --- | --- |
| **Ultrasound guidance** |  |  |
| Failure on the first attempt | 0.164 (0.012) | [3] |
| Failure on the second attempt | 0.049 (0.010) | [3] |
| Total complications on the first attempt | 0.029 (0.005) | [3] |
| Total complications on the second attempt | 0.041 (0.006) | [3,9] |
| Total complications on the third attempt | 0.047 (0.007) | [3,9] |
| Arterial puncture* | 0.015 (0.003) | [3] |
| **Landmark method** |  |  |
| Failure on the first attempt | 0.376 (0.016) | [3] |
| Failure on the second attempt | 0.166 (0.017) | [3] |
| Total complications on the first attempt | 0.114 (0.010) | [3] |
| Total complications on the second attempt | 0.156 (0.012) | [3,9] |
| Total complications on the third attempt | 0.177 (0.013) | [3,9] |
| Arterial puncture* | 0.081 (0.007) | [3] |

*SE* standard error. * The probabilities of an arterial puncture relate to each additional attempt.
