# Supplementary material for: Cost-effectiveness-analysis of ultrasound guidance for central venous catheterization compared with landmark method: a decision-analytic model
Source: BMC Anesthesiol. 2019 Apr 9;19:51. doi: 10.1186/s12871-019-0719-5 (PMC6456944; doi:10.1186/s12871-019-0719-5)
Supplement: Supplementary file 4 — Tornado-diagram presenting the results of the univariate deterministic sensitivity analysis (i.e., top ten parameters with the greatest impact on incremental cost-effectiveness ratio). (PPTX 66 kb) [file 12871_2019_719_MOESM4_ESM.pptx]

## Slide 1
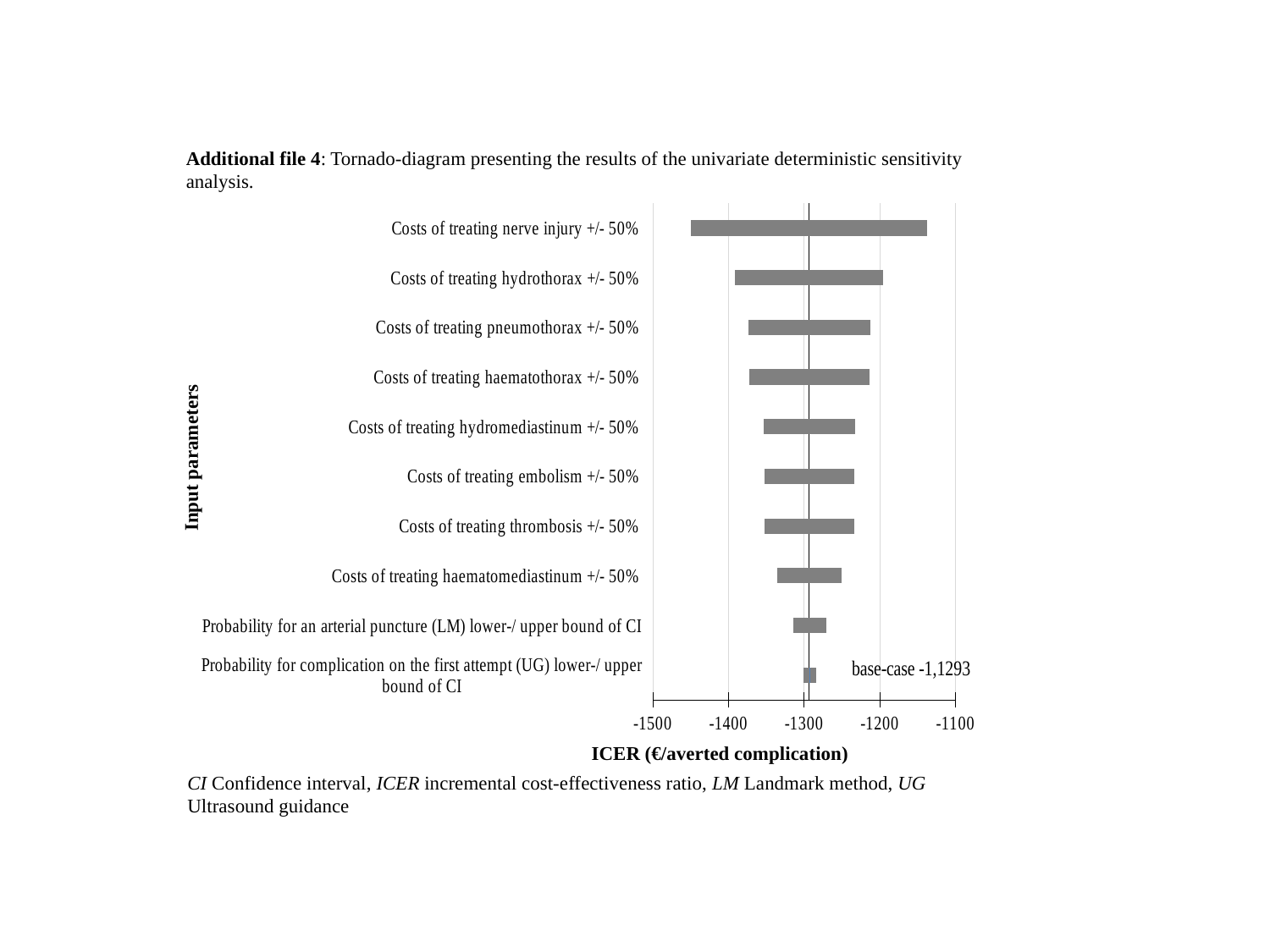

Additional file 4: Tornado-diagram presenting the results of the univariate deterministic sensitivity analysis.
Input parameters
[unsupported chart]
ICER (€/averted complication)
CI Confidence interval, ICER incremental cost-effectiveness ratio, LM Landmark method, UG Ultrasound guidance
