# Supplementary material for: Cost-effectiveness-analysis of ultrasound guidance for central venous catheterization compared with landmark method: a decision-analytic model
Source: BMC Anesthesiol. 2019 Apr 9;19:51. doi: 10.1186/s12871-019-0719-5 (PMC6456944; doi:10.1186/s12871-019-0719-5)
Supplement: Supplementary file 5 — Results of the structural sensitivity analyses presenting the output of a probabilistic parameter variation. (PPTX 620 kb) [file 12871_2019_719_MOESM5_ESM.pptx]

## Slide 1
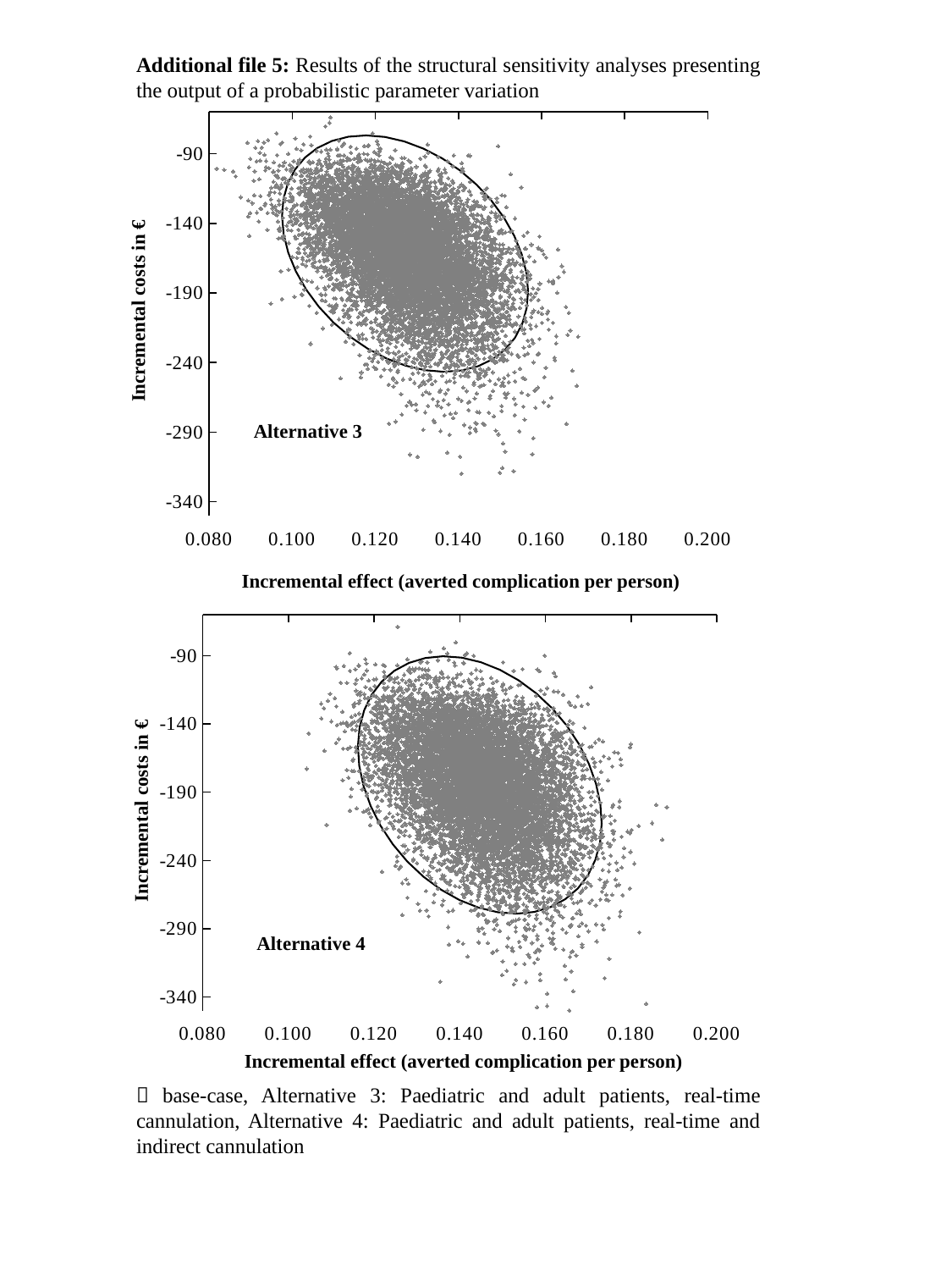

Additional file 5: Results of the structural sensitivity analyses presenting the output of a probabilistic parameter variation
### Chart
| Category | | | |
|---|---|---|---|Incremental costs in €
Incremental effect (averted complication per person)
Alternative 3
### Chart
| Category | | | |
|---|---|---|---|Incremental costs in €
Incremental effect (averted complication per person)
Alternative 4
 base-case, Alternative 3: Paediatric and adult patients, real-time cannulation, Alternative 4: Paediatric and adult patients, real-time and indirect cannulation
