# Supplementary material for: Cost-effectiveness-analysis of ultrasound guidance for central venous catheterization compared with landmark method: a decision-analytic model
Source: BMC Anesthesiol. 2019 Apr 9;19:51. doi: 10.1186/s12871-019-0719-5 (PMC6456944; doi:10.1186/s12871-019-0719-5)
Supplement: Supplementary file 6 — Results of the threshold analysis for intervention costs of ultrasound guidance. (PPTX 70 kb) [file 12871_2019_719_MOESM6_ESM.pptx]

## Slide 1
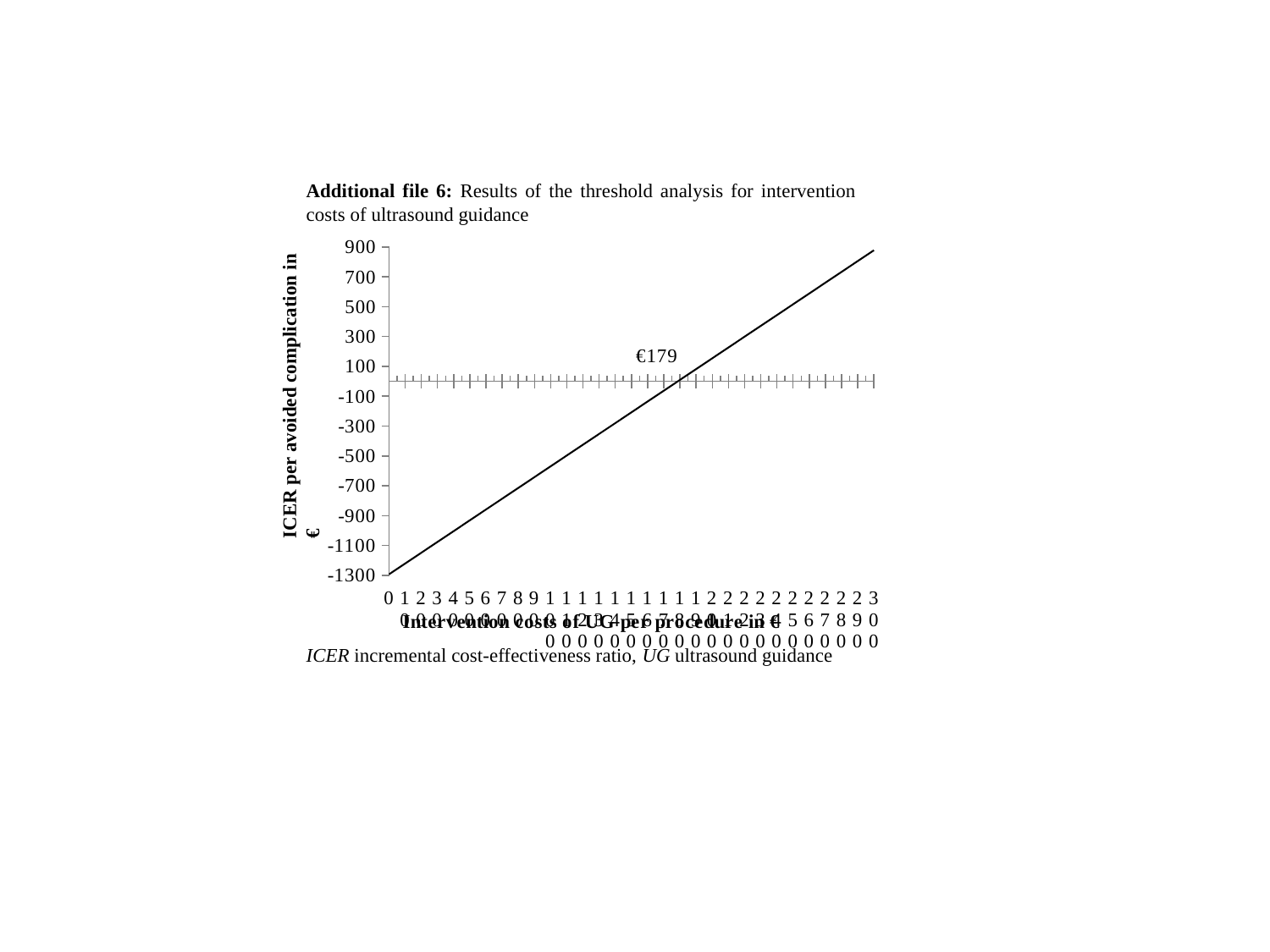

Additional file 6: Results of the threshold analysis for intervention costs of ultrasound guidance
[unsupported chart]
ICER per avoided complication in €
ICER incremental cost-effectiveness ratio, UG ultrasound guidance
